# Mesenchymal stem cells overexpressing interleukin-10 prevent allergic airway inflammation

**DOI:** 10.1186/s13287-023-03602-2

**Published:** 2023-12-13

**Authors:** Peng-Peng Kuang, Xiao-Qing Liu, Chan-Gu Li, Bi-Xin He, Ying-Chun Xie, Zi-Cong Wu, Cheng-Lin Li, Xiao-Hui Deng, Qing-Ling Fu

**Affiliations:** 1 Otorhinolaryngology Hospital, The First Affiliated Hospital, Sun Yat-sen University, 58 Zhongshan Road II, Guangzhou, 510080 Guangdong People’s Republic of China; 2 Division of Allergy, The First Affiliated Hospital, Sun Yat-sen University, Guangzhou, People’s Republic of China; 3 Extracellular Vesicle Research and Clinical Translational Center, The First Affiliated Hospital, Sun Yat-sen University, Guangzhou, 510080 People’s Republic of China

**Keywords:** Interleukin-10, Mesenchymal stem cells, Allergic airway inflammation, Immunomodulation

## Abstract

**Backgrounds:**

Allergic airway inflammation is prevalent worldwide and imposes a considerable burden on both society and affected individuals. This study aimed to investigate the therapeutic advantages of mesenchymal stem cells (MSCs) overexpressed interleukin-10 (IL-10) for the treatment of allergic airway inflammation, as both IL-10 and MSCs possess immunosuppressive properties.

**Methods:**

Induced pluripotent stem cell (iPSC)-derived MSCs were engineered to overexpress IL-10 via lentiviral transfection (designated as IL-10-MSCs). MSCs and IL-10-MSCs were administered intravenously to mice with allergic inflammation induced by ovalbumin (OVA), and the features of allergic inflammation including inflammatory cell infiltration, Th cells in the lungs, and T helper 2 cell (Th2) cytokine levels in bronchoalveolar lavage fluid (BALF) were examined. MSCs and IL-10-MSCs were co-cultured with CD4^+^ T cells from patients with allergic rhinitis (AR), and the levels of Th2 cells and corresponding type 2 cytokines were studied. RNA-sequence was performed to further investigate the potential effects of MSCs and IL-10-MSCs on CD4^+^ T cells.

**Results:**

Stable IL-10-MSCs were established and characterised by high IL-10 expression. IL-10-MSCs significantly reduced inflammatory cell infiltration and epithelial goblet cell numbers in the lung tissues of mice with allergic airway inflammation. Inflammatory cell and cytokine levels in BALF also decreased after the administration of IL-10-MSCs. Moreover, IL-10-MSCs showed a stronger capacity to inhibit the levels of Th2 after co-cultured with CD4^+^ T cells from patients with AR. Furthermore, we elucidated lower levels of IL-5 and IL-13 in IL-10-MSCs treated CD4^+^ T cells, and blockade of IL-10 significantly reversed the inhibitory effects of IL-10-MSCs. We also reported the mRNA profiles of CD4^+^ T cells treated with IL-10-MSCs and MSCs, in which IL-10 played an important role.

**Conclusion:**

IL-10-MSCs showed positive effects in the treatment of allergic airway inflammation, providing solid support for the use of genetically engineered MSCs as a potential novel therapy for allergic airway inflammation.

**Supplementary Information:**

The online version contains supplementary material available at 10.1186/s13287-023-03602-2.

## Background

Allergic airway inflammation, including allergic rhinitis (AR) and asthma, is widely distributed worldwide and is a significant burden on individuals and society [1]. Although bronchodilators and inhaled corticosteroids are widely used to suppress inflammation and relieve symptoms, they do not reverse the ongoing remodelling process. Allergen-specific immunotherapy is an effective treatment for common allergic conditions [2]. However, there are still some limitations, such as the risk of potential side effects including systemic allergic reactions. Therefore, novel therapeutic strategies are urgently needed.

Interleukin-10 (IL-10) is a soluble anti-inflammatory cytokine produced by macrophages, regulatory T cells, and other cell types that regulate the immune response and prevent tissue damage [3, 4]. Previous studies have shown that IL-10 exerts anti-inflammatory effects in various diseases including traumatic brain injury [5], stroke [6], spinal cord injury [5], and obesity [7]. IL-10 also plays a critical role in controlling allergic airway inflammation [8]. Compared to non-asthmatics, there are low levels of IL-10 in the bronchoalveolar lavage fluid (BALF) of patients with asthma [9] and low production of IL-10 from alveolar macrophages [10]. Similarly, IL-10^–/–^ mice develop enhanced allergic responses to various allergens [11–13] and exhibit increased airway eosinophilic inflammation. These studies highlight the key role of IL-10 in allergen-induced airway diseases and raise the possibility of using IL-10 as a therapeutic agent against allergic airway inflammation. However, the IL-10 protein has a short half-life of only 1–2 min in vivo, making it difficult to maintain an effective dose in the clinic.

Mesenchymal stem cells (MSCs) are multipotent stem cells not only have the ability of self-replication, but also display a broad and significant immunomodulatory role in a variety of diseases [14]. It has been reported that MSCs can migrate into damaged tissues under the stimulation of various inflammatory cytokines and release various cytokines [15] to exert therapeutic effects [16]. Our previous study showed that MSCs derived from induced pluripotent stem cells (iPSCs) inhibited the differentiation of human monocyte-derived dendritic cells (DCs) by producing IL-10 and induced the generation of IL-10-producing regulatory DCs during maturation [17]. Importantly, we reported that iPSC-MSCs exhibited a higher proliferation rate with less cell senescence, even after passage 50 [17], suggesting the possibility of undergoing genetic engineering, such as upregulating the expression of IL-10. Combined therapy with MSCs and IL-10 has been shown to reduce inflammation in collagen-induced arthritis [18] and experimental autoimmune myocarditis [19]. However, there is a paucity of research addressing whether genetically engineered MSCs expressing IL-10 can alleviate allergic airway inflammation.

The present study aimed to investigate whether treatment with MSCs engineered to overexpress IL-10 can reduce inflammation, exhibit pro-immunomodulatory effects, and improve functional recovery in allergic airway inflammation.

## Materials and methods

### Subject

Blood samples were collected from the patients with AR (*n* = 19) at The First Affiliated Hospital, Sun Yat-sen University. The eligibility criteria for patients with AR were determined based on the guidelines of the Initiative on Allergic Rhinitis and Its Impact on Asthma: (1) history of nasal symptoms due to nose itching, obstruction, sneezing, and rhinorrhea and (2) positive specific IgE. Patients were excluded from the study if they were pregnant, had received antihistamines or intranasal steroid treatment within the past month, or had taken oral steroids within the past three months. This study was approved by The Ethics Committee of The First Affiliated Hospital of Sun Yat-sen University, and informed consent was obtained from all participants.

### Animal

Female BALB/c mice (16–20 g), aged 4–6 weeks (*n* = 34), were purchased from GemPharmatech (Nanjing, China) and maintained in the specific pathogen-free Animal Experimental Centre of the North Campus, Sun Yat-sen University. All procedures involving animals were performed according to the guidelines for animal experiments and approved by the Ethics Committee of Sun Yat-sen University.

### Preparation of human iPSC-MSCs

Human iPSC-MSCs used in this study were prepared and identified as our previous report [17]. Briefly, iPSCs were reprogrammed from human urine-derived cells via electroporation with the plasmid of pEP4EO2SET2K, which contained OCT4, SOX2, SV40LT, and KLF4. The generated iPSC colonies were maintained in mTeSR1 (Stem Cell Technologies, Vancouver, Canada) supplemented with 50 U/mL of penicillin G and 50 mg/mL of streptomycin (Gibco, Invitrogen Corporation, Carlsbad, CA) until reaching 60% confluency. Then, they were induced to differentiate into MSCs using a medium containing 90% Minimum Essential Medium Eagle-α modified (α-MEM, Gibco), 10% serum replacement (Stem Cell Technologies, Vancouver, Canada), 1% penicillin/streptomycin, 1 mM sodium pyruvate, 10 mM L-ascorbate-2-phosphate (Sigma-Aldrich, Inc., St. Louis, MO), L-glutamine, and non-essential amino acids. The generated iPSC-MSCs were cultured in Dulbecco’s modified Eagle medium (DMEM; Gibco, Carlsbad, CA) supplemented with 15% foetal bovine serum (FBS; Gibco, Carlsbad, CA), 1% penicillin/streptomycin (Gibco, Carlsbad, CA), 5 ng/mL EGF (PeproTech, Rocky Hill, NJ) and 5 ng/mL β-FGF (PeproTech, Rocky Hill, NJ) at 37 °C, 5% CO_2_ and 95% humidity. Osteogenic, chondrogenic, and adipogenic differentiation capacities of iPSC-MSCs have been confirmed [17]. iPSC-MSCs in passages 16–20 were used in this study.

### Establishment of IL-10-MSCs

**Fig. 1:**
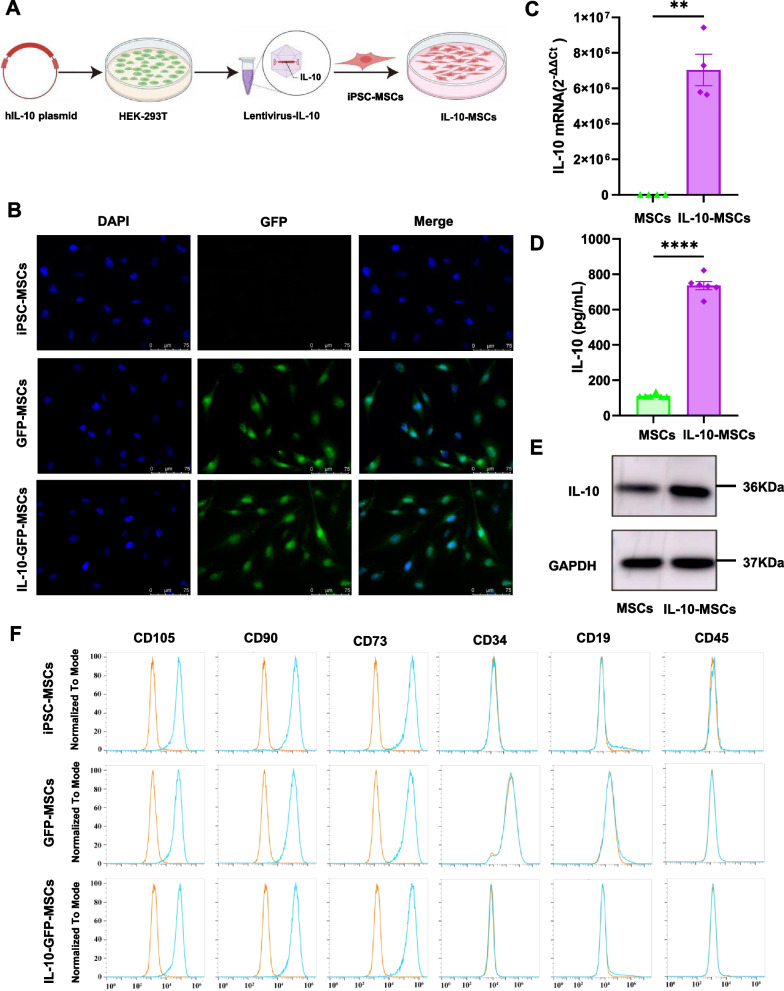
Stable IL-10-MSCs were established. **A** Protocol of establishing IL-10-MSCs. **B** GFP expressions in MSCs and IL-10-MSCs. **C** mRNA levels of IL-10 in MSCs and IL-10-MSCs. **D** IL-10 levels in the supernatant of MSCs and IL10-MSCs. **E** IL-10 protein in MSCs and IL10-MSCs. Full-length blots are presented in Additional file 1: Supplementary Fig. 2. **F** Expression of surface markers of MSCs and IL-10-MSCs. MSCs, mesenchymal stem cells; MW, molecular weight. Data are presented as mean ± SEM. ** *P* < 0.01, **** *P* < 0.0001

Human IL-10 gene (Ref. sequence NM_000572.2) plasmid was synthesised by Tsingke Biological Co. LTD (Guangzhou, China) and confirmed by sequencing. The human IL-10 ORF was inserted into a lentiviral vector (pCDH-CMV-MCS-EF1-CopGFP-T2A-Puro) without a tag. HEK-293T cells were transfected with packaging, envelope, and target plasmids using Lipofectamine 3000 (Thermo Fisher Scientific). Lentivirus-containing supernatant was collected at 48 and 72 h, and iPSC-MSCs were infected with lentivirus and selected using puromycin (2 μg/mL) to obtain stable cells with IL-10 expressing (Fig. 1A).

### Mouse model of allergic airway inflammation

**Fig. 2:**
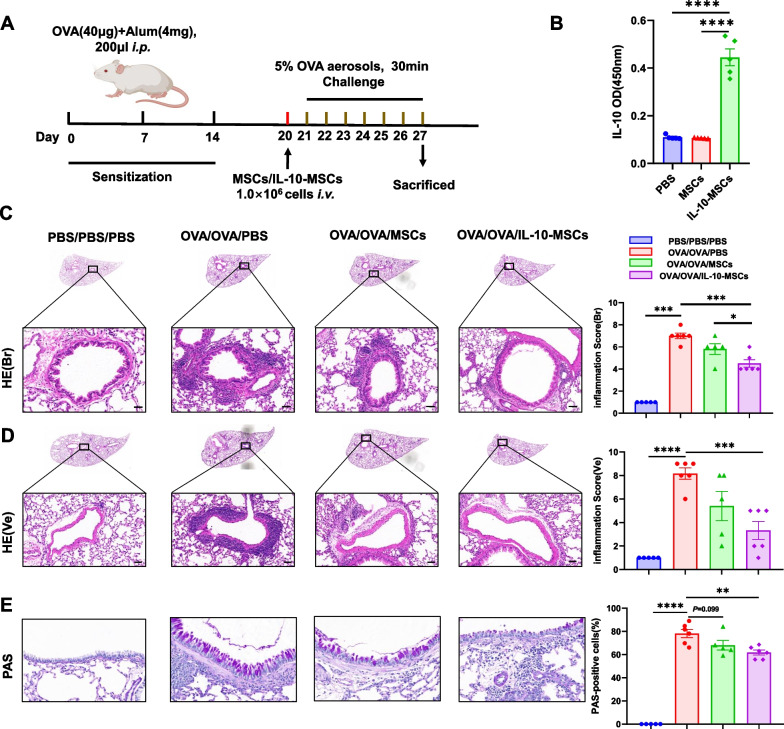
The effects of IL-10-MSCs in eosinophilic allergic airway inflammation in mice. **A** Schematic diagram for the mouse model of eosinophilic airway inflammation and the administration of MSCs or IL-10-MSCs. **B** IL-10 levels in serum 24 h after intravenous injection of PBS (*n* = 5), MSCs (*n* = 5), and IL-10-MSCs in normal mice (*n* = 5). **C–E** Representative figures and statistical results of haematoxylin and eosin (H&E) and PAS staining for lung tissues. BALF, bronchoalveolar lavage fluid; HE (Br), H&E-stained bronchia; HE (Ve), H&E-stained vessels; MSCs, Mesenchymal stem cells; OVA, Ovalbumin; PAS, Periodic acid–Schiff. Data are shown as mean ± SEM.*P < 0.05, **P < 0.01, ***P < 0.001, ****P < 0.0001. *N* = 5 for PBS/PBS/PBS and OVA/OVA/MSCs, *n* = 6 for OVA/OVA/PBS and OVA/OVA/IL-10-MSCs

A mouse model of allergic airway inflammation was established as described previously [20]. Briefly, mice were sensitised by intraperitoneal injection of 40 µg of ovalbumin (OVA, grade V, Sigma) in 4 mg of aluminium hydroxide (Thermo Fisher, 77161) in 200 µl phosphate-buffered saline (PBS) or PBS on days 0, 7, and 14, as shown in Fig. 2A. From days 21 to 27, the mice were challenged daily with aerosolised 5% OVA in a Plexiglas chamber through an air-compressing nebuliser (403A, Yuyue, China) for 30 min. MSCs or IL-10-MSCs were suspended in sterile PBS at a density of 5.0 × 10^6^ cells/mL. The mice were administrated with 1.0 × 10^6^ (200 μL) MSCs, IL-10-MSCs or the same volume of PBS via the tail vein on day 20. The control mice were treated with the same volume of PBS during the progresses of sensitisation, challenge, and treatment. The mice were randomly assigned using a random number generator to different groups: (1) PBS/PBS/PBS mice (*n* = 5) that were sensitised and challenged with PBS, followed by treatment with PBS on day 20; (2) OVA/OVA/PBS mice (*n* = 6) that were sensitised and challenged with OVA, then treated with PBS on day 20; (3) OVA/OVA/MSCs mice (*n* = 5) that were sensitised and challenged with OVA and then treated with MSCs on day 20; and (4) OVA/OVA/IL-10-MSCs mice (*n* = 6) that were sensitised and challenged with OVA and then treated with IL-10-MSCs on day 20. On day 27, 4 h after the last challenge, mice were euthanized by cervical dislocation after being anaesthetised with isoflurane (dose: 1.5%, flow rate 300 mL/min). The BALF was collected and centrifuged at 400×*g* for 5 min. The supernatants of the BALF were collected for the detection of IL-5 and IL-13, and the pellets were used for flow cytometry analysis of inflammatory cells. Left lung lobes were collected for histopathological evaluation. The remaining lung tissues were minced and digested for the isolation of lung cells following the procedures described in our previous report [21] and used for flow cytometry analyses of T helper 2 cells (Th2).

For another experiment to evaluate levels of IL-10 in mouse serum, mice were transplanted with MSCs, IL-10-MSCs, or PBS via the tail vein, and their blood was collected 24 h later through the eye enucleation method under anaesthesia with isoflurane (dose: 1.5%, flow rate 300 mL/min). The mice were then euthanized using cervical dislocation. The collected blood was centrifuged at 700×*g* for 8 min at 4 °C, and the levels of IL-10 in the serum were determined using enzyme-linked immunosorbent assay (ELISA) analysis.

### Induction of human Th2 cells and co-culture with IL-10-MSCs

Human peripheral blood mononuclear cells (PBMCs) were isolated from patients with AR. Subsequently, CD4^+^ T cells were purified by magnetic positive selection using MACS CD4 beads (Miltenyi Biotec, Bergisch Gladbach, Germany). To study the effects of IL-10-MSCs on the differentiation of Th2 cells, a total number of 2 × 10^5^ CD4^+^ T cells were seeded in 24-well plate and co-cultured with or without 2 × 10^4^ MSCs or IL-10-MSCs in Th2 polarising medium of IL-2 (10 ng/mL), IL-4 (25 ng/mL), anti-CD3 (1 µg/mL), and anti-CD28 (1 µg/mL). After 5 d, supernatants were collected for analysis of cytokine levels using ELISA, and T cells were collected for flow cytometry.

For the transwell culture experiments, iPSC-MSCs and IL-10-MSCs (5 × 10^4^ cells/well) were plated into the lower chambers of 24-well transwell plates (Costar, Corning, NY, USA), with PBMCs (5 × 10^5^ cells/well) cultured in the upper chamber. To investigate the role of IL-10 on the immunomodulatory effects of IL-10-MSCs, an anti-IL-10 monoclonal antibody (0.075 μg/mL; R&D Systems Europe) was used for the co-culture systems. After culturing for 3 d, the supernatants in the transwell were collected for ELISA analysis.

### Flow cytometric analysis

#### Determination of surface markers on MSCs

For the characterisation of surface MSCs markers, a total of ~ 3 × 10^5^ cells were harvested and resuspended in 50 μL PBS, followed by incubation with the monoclonal antibodies CD34-PE, CD45-APC, CD19-cy5.5, CD73-APC, CD90-APC, and CD105-APC. After incubation at 4 °C for 25 min, cells were washed and resuspended in washing buffer for flow cytometric analysis (BD FACSAria, NJ, USA). FlowJo V10 software was used for data analysis.

#### Flow cytometry analysis of BALF

For analysis of inflammatory cells in mouse BALF, the pellets of BALF were resuspended with 200 µL of sterile PBS, in which 50 μL cell suspension was used for cell counting using flow cytometry, and the rest 150 µL cell suspension was used for flow cytometry staining with antibodies to CD45-FITC, CD11b-APC-Cy7, CD64-PE, Ly-6G-Alexa 700, Siglec-F-Alexa 647, Ly-6C-PE-Cy7 following the gating strategies (Additional file 1: Supplementary Fig. 3) as reported previously [20]. The total number of cells in BALF was draw from the number derived from flow cytometry, and the cell numbers of eosinophils and neutrophils were gotten by the ratio of eosinophils and neutrophils.

#### Flow cytometry analysis of T helper cells in lung tissues

Lung tissues were minced and incubated in D-Hank’s buffer containing 1 mg/mL collagenase type IA (Life Technologies, Carlsbad, CA, USA) and 50 μg/mL DNaseI (Sigma, St. Louis, MO, USA) at 37 °C for 1 h. The digested lung tissues were passed through 70-µm strainers (Fisher Scientific, Pittsburgh, PA, USA) to obtain a cell suspension. Single lung cells were obtained and stimulated with phorbol myristate acetate (50 ng/mL; Sigma), ionomycin (1000 ng/mL, Sigma), and brefeldin A for 5 h. The cells were first stained with anti-CD4-PerCP-Cy5.5 (Biolegend, San Diego, CA, USA), Fixable Viability Dye-eF506 and then stained with anti-IFN-γ-PE, anti-IL-4-APC (e-Bioscience, San Diego, CA, United States). The cells were analysed using a flow cytometer (Beckman Coulter Gallios, Fullerton, CA, USA) following the gating strategies of Additional file 1: Fig. 4.

#### Flow cytometry analysis of CD4^+^ T cell

CD4^+^ T cells of AR were first stained with anti-CD4-PerCP-Cy5.5 (Biolegend, San Diego, CA, USA) and Fixable Viability Dye-eF506. CD4^+^ T cells were stimulated with phorbol myristate acetate, ionomycin, and brefeldin for 5 h, and then, the cells were fixed, permeabilised, and stained with anti-IFN-γ-PE, anti-IL-4-APC (e-Bioscience, San Diego, CA, United States). After staining, the cells were analysed using a flow cytometer (Beckman Coulter Gallios, Fullerton, CA, USA) following the gating strategies of Additional file 1: Supplementary Fig. 5A.

### CD4^+^ T cell RNA sequence

After co-cultured with MSCs or IL-10-MSCs for 5 d, human CD4^+^ T cells were purified by magnetic positive selection using MACS CD4 beads (Miltenyi Biotec, Bergisch Gladbach, Germany). The control CD4^+^ T cells, and CD4^+^ T cells co-cultured with MSCs or IL-10-MSCs were referred to as “ctrl-CD4^+^ T”, “MSCs-CD4^+^ T” and “IL-10-MSCs-CD4^+^ T”, respectively. Total RNA was extracted from the three groups using TRIzol® Reagent (Magen) according to the manufacturer’s instructions. Only qualified samples were used for library construction. Paired-end libraries were prepared using an ABclonal mRNA-seq Lib Prep Kit (ABclonal, China), following the manufacturer’s instructions. Library preparations were sequenced on an Illumina Novaseq 6000 (or MGISEQ-T7), and 150 bp paired-end reads were generated. All bioinformatics analyses were performed using an in-house pipeline from Shanghai Applied Protein Technology.

### Extraction of RNA and RT-quantitative PCR

Briefly, total RNA was extracted from MSCs and IL-10-MSCs using the RNA Quick Purification Kit (ESScience, RN001). Complementary DNA (cDNA) was synthesised using the PrimeScript RT Master Mix (Takara Bio Inc., Japan). Quantitative PCR was performed using a FastStart Universal SYBR Green Master Kit (Roche, Mannheim, Germany) to detect the expression of *IL-10*. β-Actin was used as an endogenous reference. We calculated fold changes in gene expression normalised to β-Actin using the ΔΔCT method with equation 2^−ΔΔCT^. The results are shown as fold changes compared to the control group. Primers designed for quantitative real-time PCR in this study are as follows: IL-10 (sense primers, 5′-GTTGTTAAAGGAGTCCTTGCTG-3′, and reverse primer, 5′-TTCACAGGGAAGAAATCGATGA-3′); β-Actin (sense primers, 5′-AGAGCTACGAGCTGCCTGAC-3′, and reverse primer, 5′-AGCACTGTGTTGGCGTACAG-3′).

#### Western blot

The levels of IL-10 in IL-10-MSCs were examined by western blot analysis. Equal amounts of each sample were loaded onto sodium dodecyl sulphate–polyacrylamide gels for electrophoresis (SDS-PAGE) gel. Proteins were separated on a 10% sodium dodecyl sulphate–polyacrylamide gel and transferred onto a polyvinylidene difluoride membrane (Merck Millipore, IPVH00010). The membranes were then washed in TBST for 3 × 10 min and blocked with 5% fat-free milk powder in TBST for 1.5 h. The diluted primary of antibodies as follows: rabbit anti-IL-10 (1:1000; Abcam, Cambridge, United Kingdom) and rabbit GAPDH (1:1000; Cell signalling, Danvers, MA) were added to the membrane and incubated overnight at 4 °C. Membranes were then incubated at 25 °C with a secondary antibody (1:4000, goat anti-rabbit IgG; Santa Cruz Biotechnology, Dallas, TX, USA) for 1 h. Finally, ECL (Merck Millipore, WBKLS0100) blotting detection reagents were used to visualise the membranes.

#### ELISA

The levels of human IL-10 (Invitrogen, 88-7106-22), IL-5 (Invitrogen, 88-7056-86), IL-13 (Invitrogen, 88-7439-88) and mouse IL-10 (Invitrogen, 88-7106-22), IL-5 (Invitrogen, 88-7054-86), IL-13 (Invitrogen, 88-7137-86) were analysed using ELISA kits according to the manufacturer’s instructions.

#### Statistical analysis

All the data were analysed using GraphPad 8.0 (GraphPad Software, La Jolla, CA, USA), and all the results were expressed as mean ± standard error of the mean (SEM). Statistical analyses were performed using unpaired or paired independent two-tailed Student’s t tests for single comparisons or one-way analysis of variance (ANOVA) with Tukey’s correction for multiple comparisons. A Kruskal–Wallis rank sum test followed by a Mann–Whitney *U* test was performed to compare data with an abnormal distribution. Statistical significance was set at *P* < 0.05.

## Results

### Establishing stable IL-10-MSC

In this study, IL-10 was overexpressed in iPSC-MSCs using lentiviral transfection to generate IL-10-MSCs (Fig. 1A). Positive clones were selected using puromycin and passaged as stable cell lines. As shown in Additional file 1: Supplementary Fig. 1A, IL-10-MSCs maintained normal growth when 2 μg/mL puromycin selected for 24 h or 48 h, and the morphology of IL-10-MSCs was similar to naive iPSC-MSCs (referred to as “MSCs”). The expression of green fluorescent protein (GFP) in the transfected cells was detected using immunofluorescence. The results showed that there was significant GFP expression on IL-10-MSCs and MSCs transfected with vector lentivirus (referred to as “GFP-MSCs”) compared to MSCs without transfection (Fig. 1B). To evaluate the efficiency of IL-10 transfection, we determined the GFP expression of MSCs and IL-10-MSCs (Passage 16) using flow cytometry. We found that more than 99% IL-10-MSCs were GFP positive, as shown in Additional file 1: Supplementary Fig. 1C. It suggests the successful lentivirus transfection of IL-10 into MSCs. Real-time PCR revealed that IL-10-MSCs expressed significantly higher levels of IL-10 mRNA (Fig. 1C). Moreover, we identified that IL-10-MSCs produced higher in IL-10 than in MSCs (Fig. 1D). Similarly, we confirmed that IL-10 was successfully overexpressed in IL-10-MSCs using western blotting (Fig. 1E). These results suggested that we significantly got the stable IL-10-MSCs. We further analysed the surface marker profiles of MSCs. As shown in Fig. 1F, both IL-10-MSCs and GFP-MSCs expressed high levels of surface markers CD73, CD90, and CD105 and low levels of surface markers CD34, CD19, and CD45, which was consistent with MSCs.

### IL-10-MSCs significantly ameliorated allergic airway inflammation in mice

To investigate the effect of IL-10-MSCs on allergic airway inflammation, an OVA-induced airway inflammation model was established, and MSCs or IL-10-MSCs were administered 1 d before the challenge (Fig. 2A). We determined that there was a higher level of IL-10 in the serum of normal mice after administration of IL-10-MSCs compared to that in the MSCs or PBS groups at 24 h (Fig. 2B). We observed enhanced lung inflammatory cell infiltration in the peribronchial and perivessel tissues, and more periodic acid–Schiff (PAS)-positive cells in the OVA/OVA/PBS group (referred to as “OVA group”) compared to the PBS/PBS/PBS group (Fig. 2C–E). However, treatment with IL-10-MSCs dramatically alleviated peribronchial and perivessel inflammation and decreased mucus secretion in hyperplastic goblet cells compared to the OVA group (Fig. 2C–E). There were lower inflammation scores in the peribronchial and perivessel tissues in the IL-10-MSCs groups compared in the OVA group (Fig. 2C–E).

**Fig. 3:**
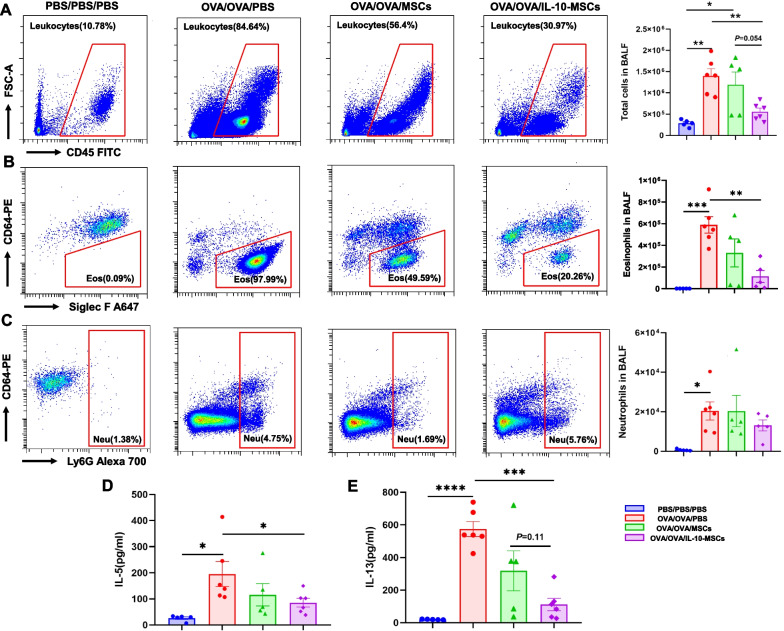
The effects of IL-10-MSCs on BALF in eosinophilic allergic airway inflammation in mice. **A–C** Inflammatory cell profiles obtained using flow cytometry in BALF. **D–E** Th2-related cytokine levels in the BALF. BALF, Bronchoalveolar lavage fluid. Data are shown as mean ± SEM. * *P* < 0.05, ** *P* < 0.01, * * * *P* < 0.001, **** *P* < 0.0001. *N* = 5 for PBS/PBS/PBS and OVA/OVA/MSCs, *n* = 6 for OVA/OVA/PBS and *n* = 5 for OVA/OVA/IL-10-MSCs (one sample was accidentally lost during the staining for eosinophils and neutrophils)

Next, we examined the effect of IL-10-MSCs on OVA-induced inflammatory cell profiles in BALF using flow cytometry. We observed increased numbers of total cells, eosinophils, and neutrophils in the BALF of OVA/OVA/PBS mice compared to PBS/PBS/PBS control mice. However, the administration of IL-10-MSCs significantly decreased the number of total inflammatory cells and eosinophils, but not neutrophils, in the BALF (Fig. 3A–C). Additionally, we observed higher levels of type 2 cytokines IL-5 and IL-13 in BALF in the OVA group, and the administration of IL-10-MSCs dramatically downregulated their levels in BALF (Fig. 3D, E).

**Fig. 4:**
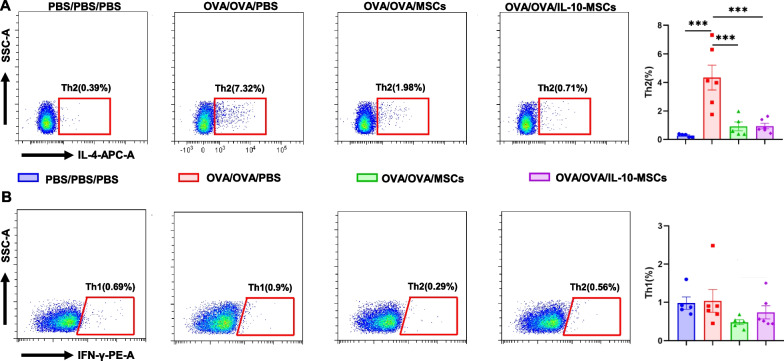
The effects of IL-10-MSCs on Th2 in eosinophilic allergic airway inflammation. **A** Th2 response in lung tissues with MSCs or IL-10-MSCs treatment. **B** Th1 response in lung tissues with MSCs or IL-10-MSCs treatment. Data are shown as mean ± SEM.* * * *P* < 0.001, **** *P* < 0.0001. *N* = 5 for PBS/PBS/PBS and OVA/OVA/MSCs, *n* = 6 for OVA/OVA/PBS and OVA/OVA/IL-10-MSCs

We also evaluated the proportion of Th2 cells in lungs. OVA/OVA/PBS mice displayed a significantly higher percentage of Th2 cells, and IL-10-MSCs significantly reduced the number of Th2 cells in the lungs (Fig. 4A). In contrast, no differences were observed in the number of Th1 cells among the three groups (Fig. 4B). Of course, here we did not find the difference for Th2 levels between MSCs and IL-10-MSCs treatments.

In summary, our above findings demonstrate that administration of IL-10-MSCs significantly prevented allergic airway inflammation in mice.

### IL-10-MSCs inhibited the Th2 response for patients with AR

**Fig. 5:**
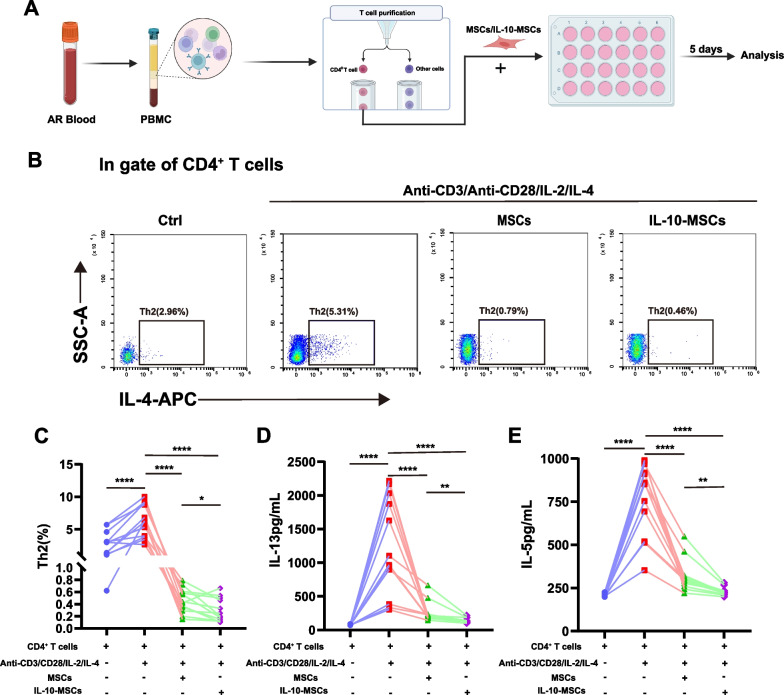
The effects of IL-10-MSCs on Th2 response in the peripheral blood of patients with AR. **A** Schematic protocol for isolation CD4^+^ T cells and co-culturing with MSCs or IL-10-MSCs. **B–C** Th2 cell levels after co-culturing with MSCs or IL-10-MSCs (*n* = 12). **D–E** IL-13 and IL-5 levels in supernatants were analysed using ELISA. Data are shown as mean ± SEM. * *P* < 0.05, * * * *P* < 0.01, * * * *P* < 0.001

Next, we investigated the immunoregulatory effects of IL-10-MSCs on T cells using PBMCs derived from patients with AR. Purified CD4^+^ T cells were polarised to Th2 cells and co-cultured with MSCs or IL-10-MSCs for 5 d (Fig. 5A). We confirmed that the purity of CD4^+^ T cells was > 98% using flow cytometry (Additional file 1: Supplementary Fig. 5B). We observed a significantly higher Th2 level under polarising stimulation; treatment with both types of MSCs significantly reversed the level of Th2 cells, and IL-10-MSCs exhibited a significantly better inhibitory effect than MSCs (Fig. 5B, C, *P* < 0.05). Similarly, we determined that the levels of IL-13 and IL-5 were significantly increased in response to polarising conditions but decreased after co-culturing with MSCs and IL-10-MSCs. Importantly, IL-13 and IL-5 levels were lower after the administration of IL-10-MSCs than after the administration of MSCs (Fig. 5D, E, *P* < 0.001). These results suggested that IL-10-MSCs exhibit a high immunoregulatory capacity for allergic inflammation.

### IL-10 was involved in the immunomodulation of IL-10-MSCs on the production of type 2 cytokines

**Fig. 6:**
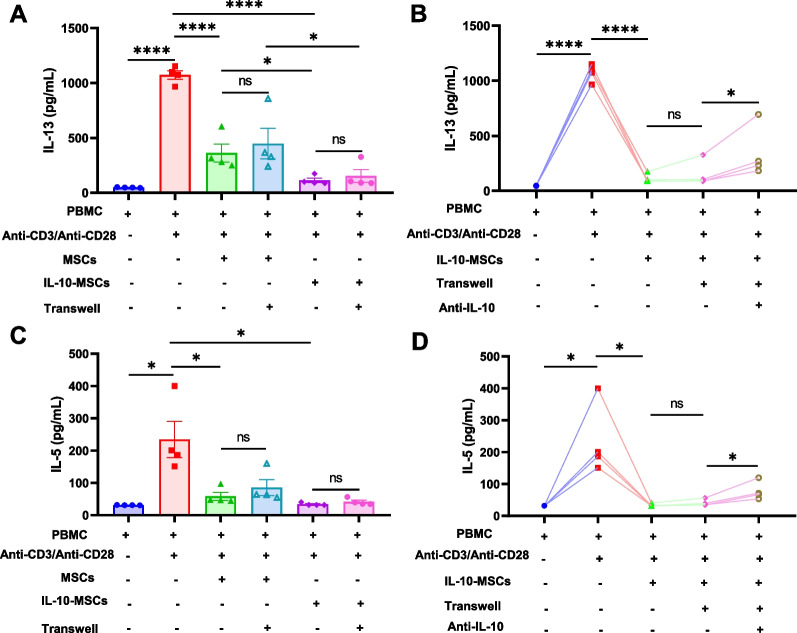
IL-10 was involved in the immunomodulation of IL-10-MSCs on IL-13 and IL-5 production. **A**, **C** Role of cell–cell contact in the effects of IL-10-MSCs on IL-13 and IL-5 production (*n* = 4); **B**, **D** Role of IL-10 in the effects of IL-10-MSCs on IL-13 and IL-5 production. Data are shown as mean ± SEM. * *P* < 0.05, ** *P* < 0.01, **** *P* < 0.0001

Next, we investigated the possible mechanisms of action of IL-10-MSCs on Th2 cells. MSCs have been reported to exert their therapeutic effects via cell–cell contact and paracrine signalling. Our previous studies showed that iPSC-MSCs exert their effects on DC differentiation through cell-to-cell contact [17] and on antigen-stimulated PBMCs from patients with AR via prostaglandin E2 [22]. Next, we investigated the role of cell-to-cell contact and IL-10 in the effects of IL-10-MSCs on type 2 cytokines using PBMCs derived from patients with AR. Similarly, the high levels of IL-13 and IL-5 in response to Anti-CD3/Anti-CD28 were significantly decreased after co-cultured with IL-10-MSCs. We did not find any difference in the levels of IL-13 after PBMCs were co-cultured with IL-10-MSCs in a transwell system (Fig. 6A), suggesting that cell–cell contact may not be involved in the immunomodulation of IL-10-MSCs in the production of type 2 cytokines. However, higher levels of IL-13 were observed after the administration of the anti-IL-10 antibody compared to IL-10-MSC treatment alone (Fig. 6B). Similar results were observed for the IL-5 levels (Fig. 6C, D). These data indicate that IL-10 plays a major role in the IL-10-MSCs-mediated inhibition of IL-13 and IL-5 production.

### The mRNA profiles for CD4^+^ T cells with the treatments of IL-10-MSCs and MSCs

**Fig. 7:**
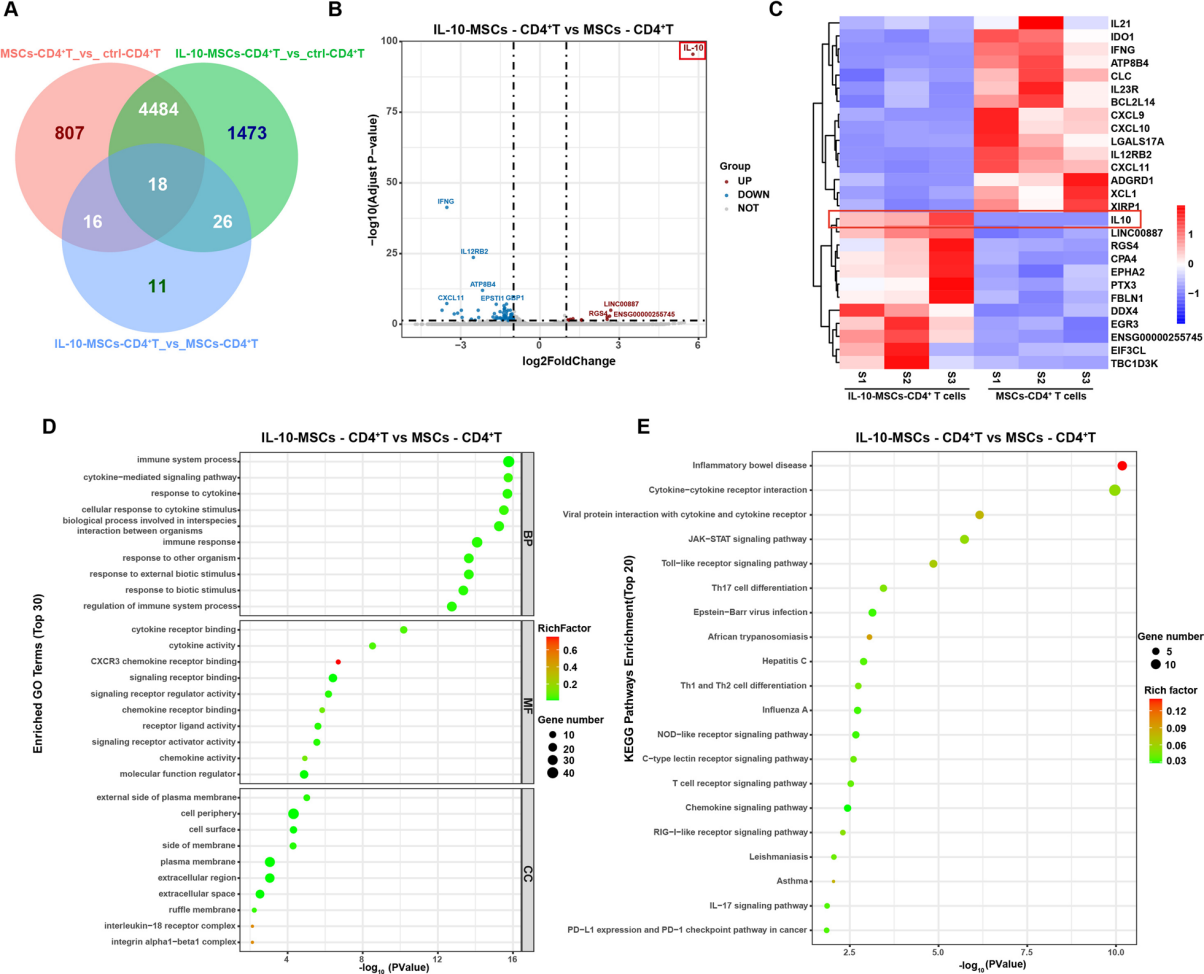
mRNA profiling of CD4^+^ T cells with MSCs treatment. **A** Venn diagram showing differentially expressed mRNAs in CD4^+^ T, MSCs-CD4^+^ T, and IL-10-MSCs-CD4^+^T cells (*n* = 3). **B** Volcano plot showing differentially expressed mRNAs in MSCs-CD4^+^ T cells and IL-10-MSCs-CD4^+^T cells. **C** Heatmap representation of differential mRNAs involved in IL-10-MSCs-CD4^+^T and MSCs-CD4^+^ T cells **D–E** GO and KEGG enrichment analyses of differential mRNAs between MSCs-CD4^+^ T and IL-10-MSCs-CD4^+^T cells

To investigate possible functional differences in CD4^+^ T cells between the MSCs and IL-10-MSCs group, we performed RNA sequencing of ctrl-CD4^+^ T cells, MSCs-CD4^+^ T cells and IL-10-MSCs-CD4^+^ T cells. We elucidated a significant difference in the mRNA profiles of CD4^+^ T cells after co-cultured with MSCs or IL-10-MSCs. In total, we identified 5325 and 6001 differentially expressed mRNAs between ctrl-CD4^+^ T cells and MSCs-CD4^+^ T cells, and ctrl-CD4^+^ T cells and IL-10-MSCs-CD4^+^ T cells, respectively. Interestingly, only 71 mRNAs were identified to express differentially between MSCs-CD4^+^ T cells and IL-10-MSCs-CD4^+^ T cells (Fig. 7A). Among the 71 differentially expressed mRNAs, 12 were upregulated and 59 were decreased in IL-10-MSCs-CD4^+^ T cells compared to those in MSCs-CD4^+^ T cells (adjusted *P* value < 0.05) (Fig. 7B). IL-10 was the most significantly upregulated mRNAs in IL-10-MSCs-CD4^+^ T cells, suggesting that IL-10 levels in CD4^+^ T cells increased in response to IL-10-MSCs treatment, which may also exert an inhibitory effect on Th2 cells. Thirty mRNAs involved in the regulation of Th2 function are shown in Fig. 7C. In addition to IL-10, EPHA2 and PTX3 were upregulated in IL-10-MSCs-CD4^+^ T cells, which have been reported to play an inhibitory role in Th2 function [23] or airway hyperresponsiveness [24]. RGS4 was found to be upregulated too. However, previous studies have shown that RGS4 promotes allergen- and aspirin-induced airway hyperresponsiveness [25]. Using Gene Ontology (GO) enrichment analysis, we determined that the differentially expressed mRNAs were associated with processes commonly associated with inflammation, such as immune responses, cytokines and their receptor signalling pathways, and receptor–ligand activity. Similarly, Kyoto Encyclopaedia of Genes and Genomes (KEGG) pathway analysis showed that cytokine–cytokine receptor interactions, the Jak-Stat signalling pathway, the chemokine signalling pathway, and Th1 and Th2 cell differentiation were involved in these mRNA changes (Fig. 7D, E). These results further suggest that treatment with IL-10-MSCs had more effects on cytokine production and interaction than MSCs.

## Discussion

In this study, we introduced the IL-10 gene into human iPSC-MSCs to establish highly overexpressing IL-10-MSCs and confirmed their strong immunomodulatory effects on allergic airway inflammation. We demonstrated that IL-10-MSCs significantly prevented allergic airway inflammation in a mouse model and decreased the function of type 2 cytokines in patients with AR. Our findings suggest a novel therapeutic strategy for allergic airway disease based on IL-10-MSCs.

MSCs have been frequently considered as candidates for immunotherapy because of the beneficial effects of MSC-based therapies in the treatment of different pathologies. The therapeutic benefits of MSCs are thought to result from their immunomodulatory and anti-inflammatory properties via soluble factors that have been thoroughly investigated for the treatment of inflammatory diseases both in vitro and in vivo [26]. IL-10 is a key cytokine involved in the control of allergic airway inflammation. In allergic asthma, where pathological responses to inhaled allergens develop due to a failure in immune tolerance, a successful therapeutic strategy is associated with an increase in IL-10 levels. This is the case for the glucocorticoid dexamethasone, which facilitates IL-10 production by human CD4^+^ and CD8^+^ T cells [27]. In addition, severe steroid-resistant asthma is associated with failure of IL-10 enhancement by patient cells in response to dexamethasone [28]. Several strategies have also been developed for enhancing IL-10 levels in the context of neurological diseases using immune components [29]. These include administration of recombinant IL-10, enhancement of IL-10 production using agonists, and delivery of IL-10 via viral vectors [4].

Because of the short half-life of recombinant IL-10, it is difficult to maintain a relatively stable concentration in the body in humans [30]. In our study, iPSC-MSCs were used as carriers to establish IL-10-MSCs. In a mouse model, the data showed that the administration of IL-10-MSCs boosted the serum level of IL-10 when compared with the MSCs or PBS group. Compared with the half-life of the IL-10 protein in vivo of 1–2 min, mice injected with IL-10-MSCs maintained higher serum levels of IL-10 even after 24 h. Furthermore, we determined that human IL-10-MSCs effectively attenuated lung pathology, decreased the levels of Th2 cytokines in BALF, and inhibited the activation of Th2 cells in the lung tissue.

Subsequently, we co-cultured CD4^+^ T cells with MSCs or IL-10-MSCs. We determined a decreased level of Th2 in CD4^+^ T cells after co-culturing with MSCs and IL-10-MSCs, and the production of IL-13 and IL-5 in Th2 cells was significantly decreased. Importantly, in our study, IL-10-MSCs demonstrated stronger inhibitory effects on T-cell differentiation than MSCs did. These results are consistent with previous reports [22, 31]. Coomes et al*.* [31] have demonstrated that IL-10 directly limits Th2 cell differentiation and survival in vitro and in vivo. Ablation of IL-10 signalling in Th2 cells enhanced Th2 cell survival and exacerbated pulmonary inflammation in a murine model of house dust mite allergy. Similarly, in the current study, the immunosuppressive effect of IL-10-MSCs was able to be specifically reversed using an anti-IL-10 monoclonal antibody. Mechanistically, we performed RNA-sequence of CD4^+^ T cells to evaluate different mRNAs related to Th2 regulation in MSCs and IL-10-MSCs. IL-10 was the most significantly upregulated mRNAs in IL-10-MSCs-CD4^+^ T cells, suggesting that IL-10 levels in CD4^+^ T cells increased in response to IL-10-MSCs treatment. The results of a study by Barrat et al*.* [27] showed that vitamin D3 and Dexamethasone induced naïve human and mouse CD4^+^ T cells to produce IL-10. Our data suggest that IL-10-MSCs treatment at least partially exerts the same effects as dexamethasone treatment. Both in vivo and in vitro, IL-10-MSCs showed better therapeutic effects than MSCs, further reinforcing the immunoregulatory effects of IL-10-MSCs.

Anti-inflammatory strategies using anti-inflammatory cytokines are promising for the treatment of allergic airway inflammation. Current MSC-based augmentation of IL-10 therapy has achieved good efficacy in a variety of diseases. Gao et al*.* [32] introduced the IL-10 gene into human umbilical cord-derived MSCs (HUCMSCs) and determined that HUCMSCs overexpressing IL-10 significantly enhanced functional recovery after spinal cord injury by directionally activating macrophages. Wang et al*.* [33] constructed HUCMSCs stably overexpressing IL-10 which were applied to obese mice, and compared with HUCMSCs, IL-10-HUCMSCs treatment had much better anti-obesity effects, including body weight reduction, greater glucose tolerance, less systemic insulin resistance, and less adipose tissue inflammation in HFD feeding mice. In addition, Peruzzaro et al*.* [34] observed a significant improvement in fine motor function in rats transplanted with bone marrow (BM)-MSCs engineered to overexpress IL-10. However, despite the availability of MSCs from the umbilical cord or bone marrow, these cells have a limited proliferative capacity, large variability in cell quality derived from different donors and quickly lose their differentiation potential [35]. All these factors limit their therapeutic benefits, especially their clinical applications. In contrast, MSCs loaded with IL-10, derived from iPSCs, were reprogrammed from human urine cells. Urine cells can be easily obtained from most people; therefore, it is a viable and non-invasive method for gathering an endless supply of human cells for reprogramming. In addition, the proliferation rate of iPSC-MSCs is higher [36]. Importantly, it has been reported that, compared to bone marrow (BM)-MSCs, human iPSC-MSCs are less immunogenic and have a stronger immune privilege after transplantation [37]. These results suggest that iPSC-MSCs are a readily available and acceptable source of MSCs, particularly for therapeutic applications. Therefore, our iPSC-MSC-based overexpression IL-10 therapy has a greater clinical translational potential.

The present study has several limitations that should be acknowledged. First, our animal experiments only utilised a single dose and a single administration of MSCs and IL-10-MSCs. However, this approach may not fully capture the dose-dependent responses and long-term effects of these treatments. To gain a more comprehensive understanding of the underlying mechanisms and optimise therapeutic approaches, it would be beneficial to conduct further studies involving varying dosages and multiple intravenous administrations. Second, our study primarily focused on the preventive effects of MSCs and IL-10-MSCs. This narrow focus limits the generalisability of the findings to situations in which IL-10-MSCs can be administered at symptom onset. To expand the therapeutic applicability of IL-10-MSCs, it would be valuable to investigate the effects of administering IL-10-MSCs after challenge. This study provides a more comprehensive understanding of the potential benefits of IL-10-MSCs in treating allergic airway inflammation. Finally, findings from animal studies may not directly translate to humans because of inherent biological differences between species. It is crucial to replicate and validate the findings of animal studies in humans.

## Conclusions

Collectively, our data suggest that iPSC-MSCs engineered to overexpress IL-10 exert better therapeutic effects in allergic airway inflammation than MSCs alone. This indicates that the upregulation of IL-10 in MSCs is useful for the treatment of allergic airway inflammation, providing new insights for cell-based therapeutic products in allergic diseases.

### Supplementary Information

**Additional file 1. Supplementary Fig. 1.** Construction and characterization of IL-10-MSCs. **A**. iPSC-MSCs transfected with IL-10 gene were selected under 2 μg/mL puromycin. **B**. plasmid backbone of IL-10 gene. C. Expression of GFP in IL-10-MSCs (P16 was used for flow cytometry detection). **Supplementary Fig. 2.** Original uncropped blots for Fig. 1E. **Supplementary Fig. 3.** Gating strategy for flow cytometry analyses of inflammatory cells in bronchoalveolar lavage fluid. **Supplementary Fig. 4.** Gating strategy for flow cytometry analyses of lung tissues. **Supplementary Fig. 5.** Gating strategy for Th2 and sort of CD4^+^ T cells. A. Gating strategy for flow cytometry analyses of Th2 cells in PBMCs. B. The sort of CD4^+^ T cells.

## Data Availability

The data that support the findings of this study are available from the corresponding author upon reasonable request. The RNA sequence datasets presented in this study can be found in online repositories. The names of the repositories and accession numbers can be found below: SRA, Accession PRJNA1005638 (https://www.ncbi.nlm.nih.gov/sra).

## Abbreviations

AR: Allergic rhinitis
BM-MSCs: Bone marrow-mesenchymal stem cells
BALF: Bronchoalveolar lavage fluid
DCs: Dendritic cells
ELISA: Enzyme-linked immunosorbent assay
GO: Gene Ontology
GFP: Green fluorescent protein
HUCMSCs: Human umbilical cord-derived MSCs
iPSCs: Induced pluripotent stem cells
IL-10: Interleukin-10
KEGG: Kyoto Encyclopaedia of Genes and Genomes
MSCs: Mesenchymal stem cells
OVA: Ovalbumin
PAS: Periodic acid–Schiff
PBMCs: Peripheral blood mononuclear cells
PBS: Phosphate-buffered saline
SEM: Standard error of the mean
SDS-PAGE: Sodium dodecyl sulphate-polyacrylamide gel electrophoresis
Th2: T helper 2 cell
