# Supplementary material for: Mesenchymal stem cells overexpressing interleukin-10 prevent allergic airway inflammation
Source: Stem Cell Res Ther. 2023 Dec 13;14:369. doi: 10.1186/s13287-023-03602-2 (PMC10720159; doi:10.1186/s13287-023-03602-2)
Supplement: Supplementary file 1 — Additional file 1. Supplementary Fig. 1. Construction and characterization of IL-10-MSCs. A. iPSC-MSCs transfected with IL-10 gene were selected under 2 μg/mL puromycin. B. plasmid backbone of IL-10 gene. C. Expression of GFP in IL-10-MSCs (P16 was used for flow cytometry detection). Supplementary Fig. 2. Original uncropped blots for Fig. 1E. Supplementary Fig. 3. Gating strategy for flow cytometry analyses of inflammatory cells in bronchoalveolar lavage fluid. Supplementary Fig. 4. Gating strategy for flow cytometry analyses of lung tissues. Supplementary Fig. 5. Gating strategy for Th2 and sort of CD4+ T cells. A. Gating strategy for flow cytometry analyses of Th2 cells in PBMCs. B. The sort of CD4+ T cells. [file 13287_2023_3602_MOESM1_ESM.docx]

**Supplementary Figures**

**
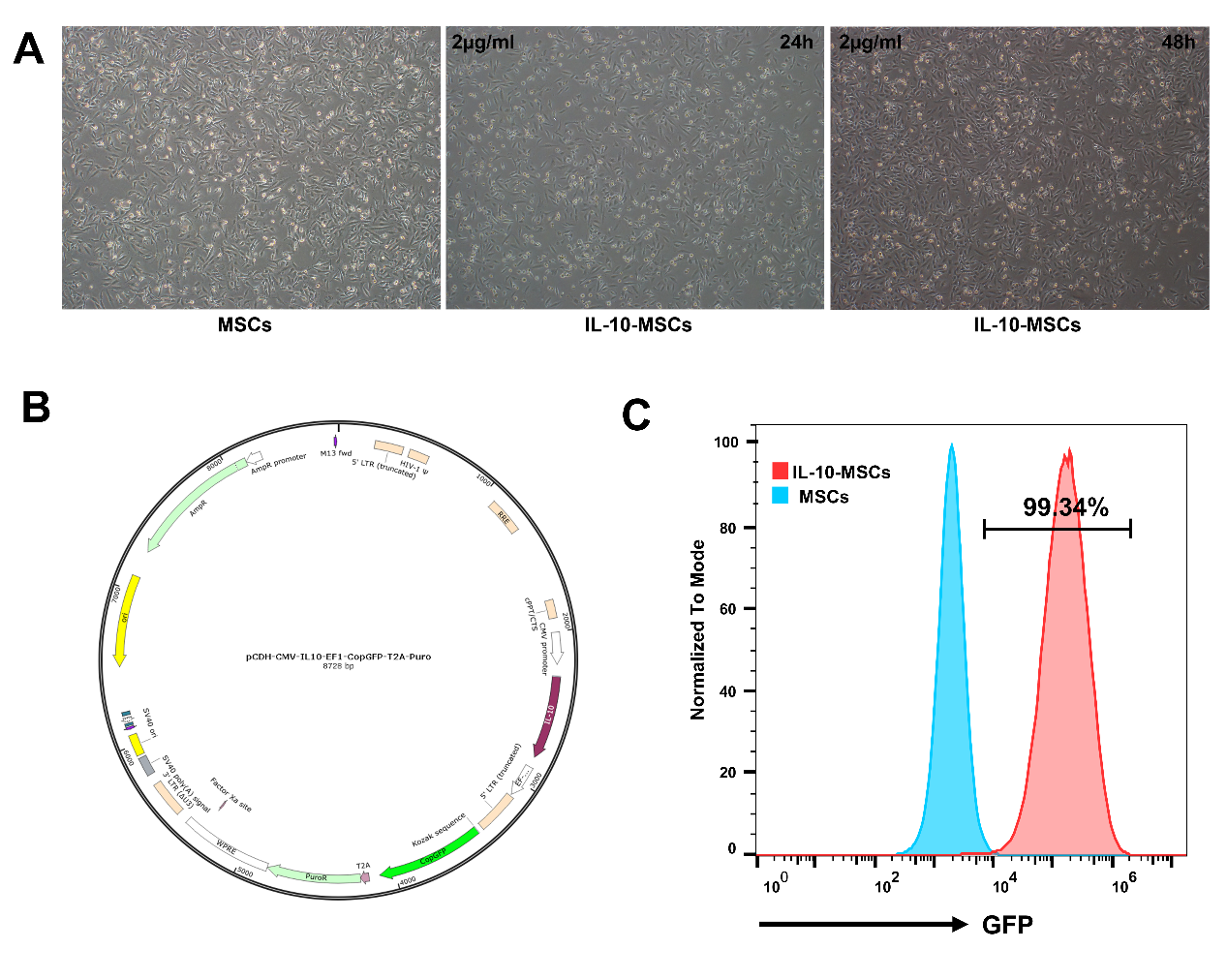
**

**Supplementary Fig.1** Construction and characterization of IL-10-MSCs. **A.** iPSC-MSCs transfected with IL-10 gene were selected under 2 μg/mL puromycin. **B**. plasmid backbone of IL-10 gene. **C.** Expression of GFP in IL-10-MSCs (P16 was used for flow cytometry detection)


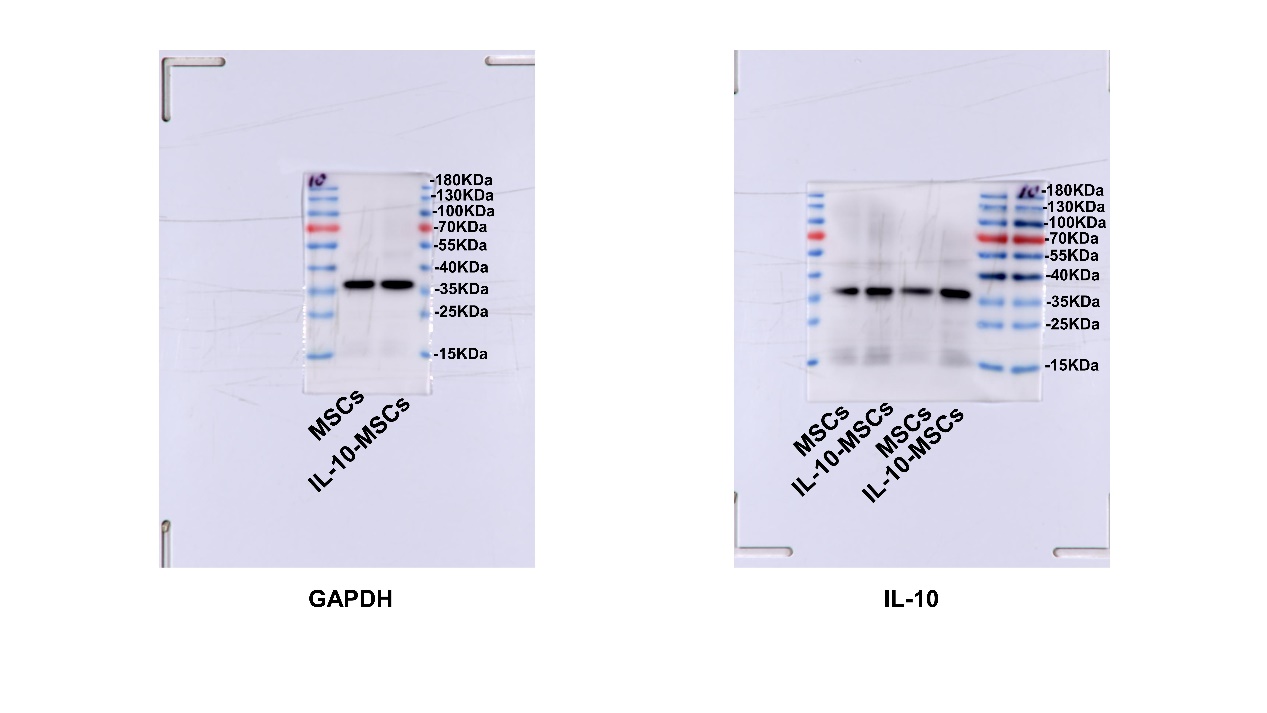
**Supplementary Fig.2** Original uncropped blots for Fig. 1E.


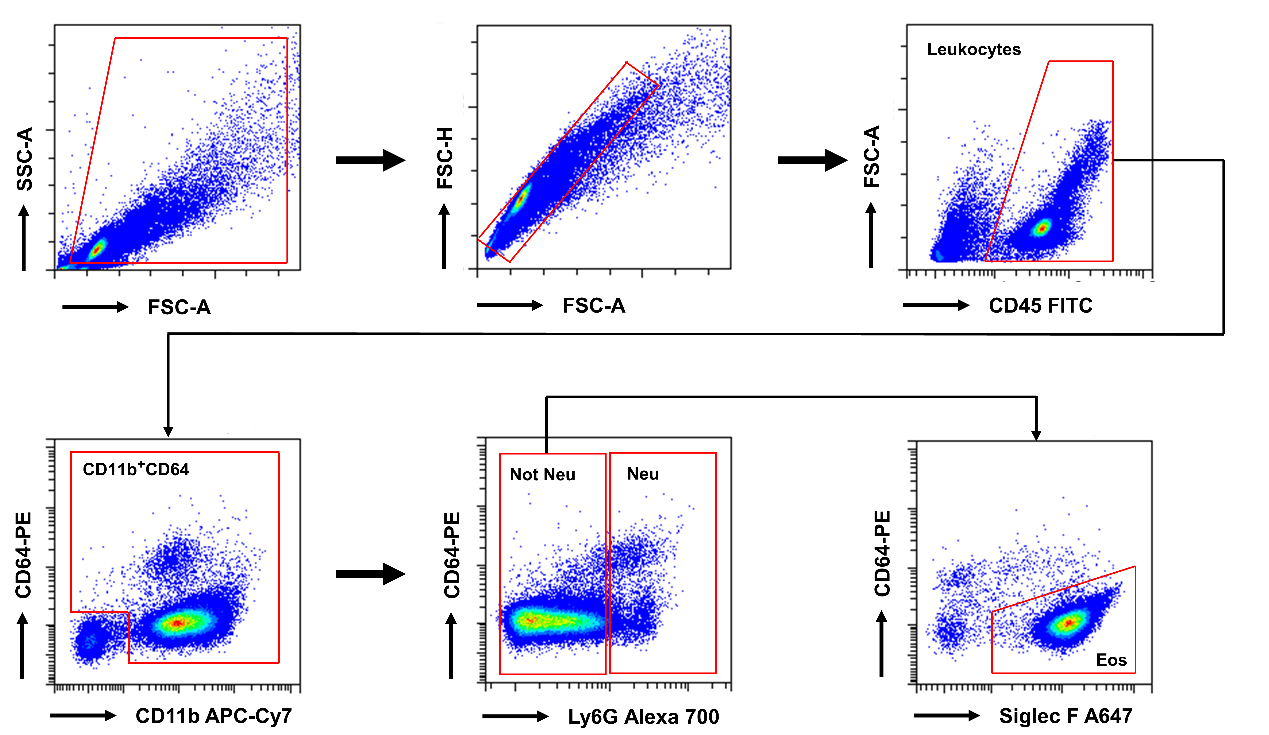


**Supplementary Fig.3** Gating strategy for flow cytometry analyses of inflammatory cells in bronchoalveolar lavage fluid.


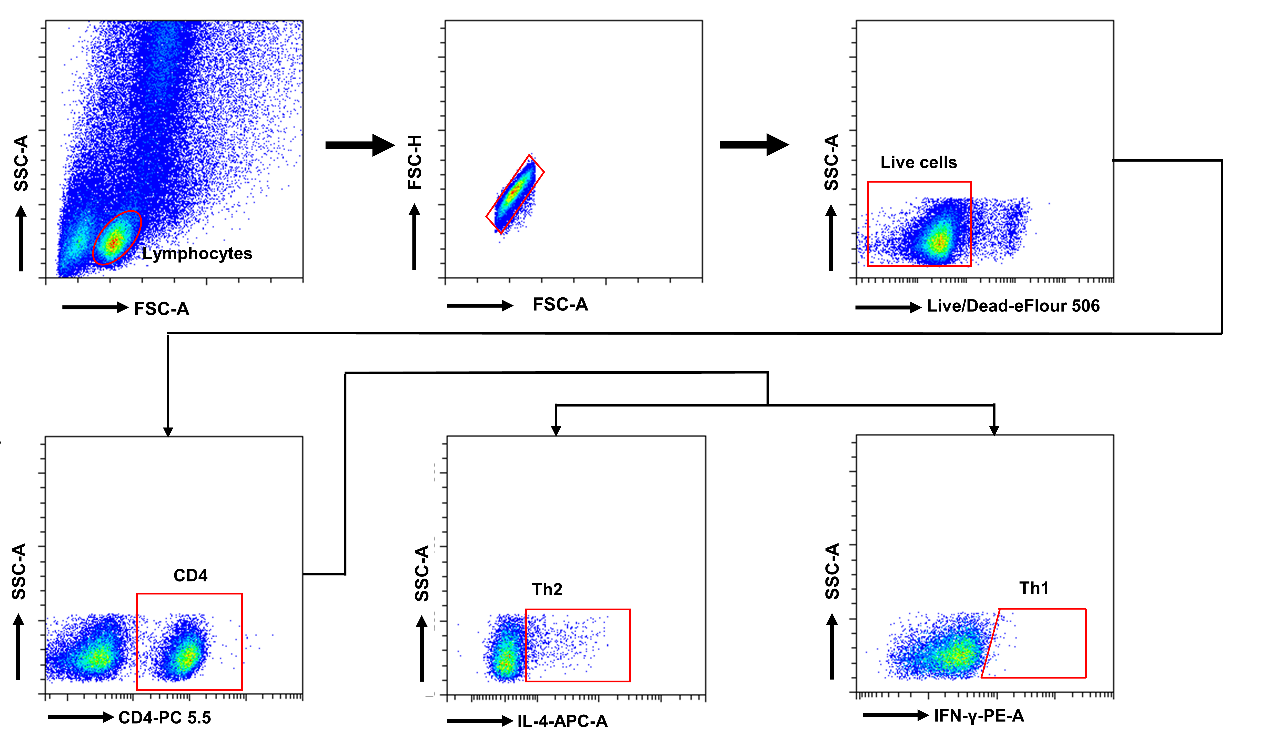


**Supplementary Fig.4** Gating strategy for flow cytometry analyses of lung tissues.


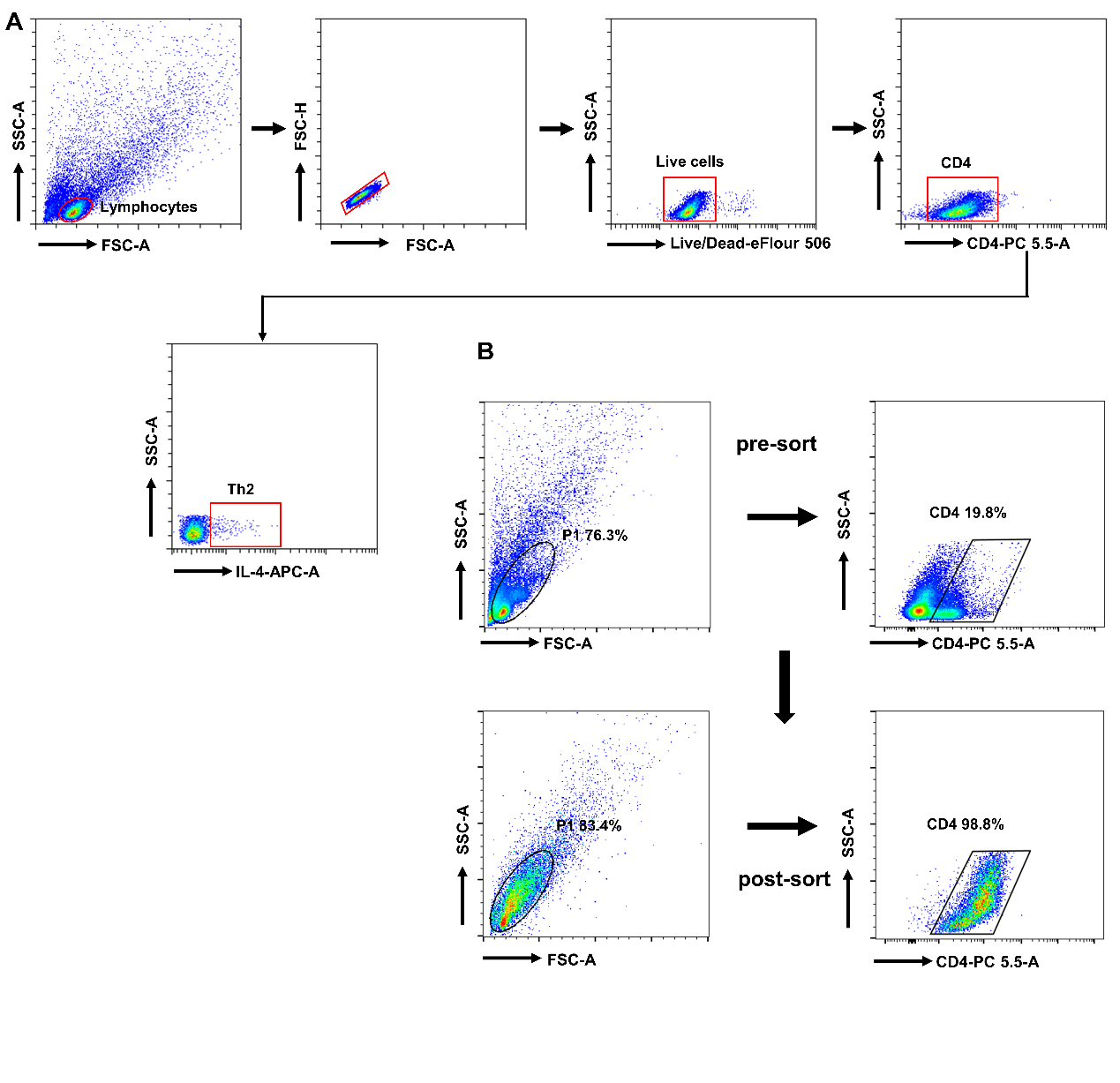


**Supplementary Fig.5** Gating strategy for Th2 and sort of CD4^+^ T cells. **A.** Gating strategy for flow cytometry analyses of Th2 cells in PBMCs. **B.** The sort of CD4^+^ T cells.
